# Effects of Fermented *Polygonum cuspidatum* on the Skeletal Muscle Functions

**DOI:** 10.3390/nu16020305

**Published:** 2024-01-19

**Authors:** Young-Seon Kim, Ji-Hye Han, Chang-Hoon Lim, Xue-Quan Fang, Hyeock-Soon Jang, Sang-Yun Lee, Woo-Jong Yim, Ji-Hong Lim

**Affiliations:** 1 Department of Medicinal Biosciences, College of Biomedical & Health Science, Konkuk University, 268, Chungwon-daero, Chungju 27478, Chungbuk, Republic of Korea; yskim0801@kku.ac.kr (Y.-S.K.); lchoo1196@kku.ac.kr (C.-H.L.); gkrrnjs654852@kku.ac.kr (X.-Q.F.); 2 BK21 Program, Department of Applied Life Science, Graduate School, Konkuk University, 268, Chungwon-daero, Chungju 27478, Chungbuk, Republic of Korea; 3 Jung-Ang Microbe Research Institute (JM), 398, Jikji-daero, Heungdeok-gu, Cheongju 28576, Chungbuk, Republic of Korea; hanjihye66@naver.com (J.-H.H.); hyeocksj@naver.com (H.-S.J.); willufine@gmail.com (S.-Y.L.); ywj0808@naver.com (W.-J.Y.); 4 Center for Metabolic Diseases, Konkuk University, 268, Chungwon-daero, Chungju 27478, Chungbuk, Republic of Korea

**Keywords:** fermentation, *Polygonum cuspidatum*, emodin, resveratrol, skeletal muscle

## Abstract

Plant extract fermentation is widely employed to enhance the nutritional and pharmaceutical value of functional foods. *Polygonum cuspidatum* (Pc) contains flavonoids, anthraquinones, and stilbenes, imparting protective effects against inflammatory diseases, cancer, diabetes, and cardiovascular diseases. However, the effects of fermented Pc on skeletal muscle strength remain unexplored. In this study, we generated fermented Pc using a complex of microorganisms containing *Lactobacillus* spp. (McPc) and assessed its effects on muscle strength and motor function in mice. Compared to unfermented Pc water extract, elevated levels of emodin and resveratrol were noted in McPc. This was identified and quantified using UPLC-QTOF/MS and HPLC techniques. Gene expression profiling through RNA-seq and quantitative RT-PCR revealed that McPc administration upregulated the expression of genes associated with antioxidants, glycolysis, oxidative phosphorylation, fatty acid oxidation, and mitochondrial biogenesis in cultured C2C12 myotubes and the gastrocnemius muscle in mice. McPc significantly improved skeletal muscle strength, motor coordination, and traction force in mice subjected to sciatic neurectomy and high-fat diet (HFD). McPc administration exhibited more pronounced improvement of obesity, hyperglycemia, fatty liver, and hyperlipidemia in HFD mice compared to control group. These findings support the notion that emodin and resveratrol-enriched McPc may offer health benefits for addressing skeletal muscle weakness.

## 1. Introduction

*Polygonum cuspidatum* (Pc) primarily comprises polydatin, resveratrol, emodin, quercetin, and their derivatives as principal bioactive phytochemical components. The therapeutic and preventive effects of Pc against various human diseases have been extensively investigated [1]. The anti-inflammatory, antioxidant, antiviral, and antimicrobial properties of Pc have been validated in cultured cell lines and animal models [1]. Moreover, the therapeutic efficacy of Pc has been assessed in conditions such as arthritis [2], ulcerative colitis [3], asthma [4], cardiac hypertrophy [5], cancer [1], and cancer-associated cachexia [6]. 

Recently, the fermentation of natural products using microorganisms has gained widespread application for extending shelf life, improving food safety and nutritional value, and enriching bioactive components [7]. Numerous health benefits of fermented blueberries using various microorganisms such as *Serratia vacinii*, *Lactobacillus plantarum*, *L. rhamnosus*, *Saccharomyces bayanus*, *Bacillus amyloliquefaciens*, *L. brevis*, and *Starmerella bombicola* have been investigated in human studies. Fermented blueberries with *Lactobacillus* spp. and *Streptococcus thermophilus* have demonstrated antidiabetic and antiobesity effects [8]. Additionally, polyphenol-enriched blueberries fermented with *Rouxiella badensis* subsp. *acadiensis* exhibited anticancer, anti-inflammatory, and antioxidant effects both in vitro and in vivo [9,10]. Despite the diverse phytochemical components and health benefits of Pc against various chronic human diseases, Pc fermentation has not been attempted. 

Skeletal muscle weakness, including mass, strength, and function, commonly referred to as sarcopenia, is linked to physical frailty and an elevated risk of chronic diseases [11]. Aging and obesity are closely associated with the loss of skeletal muscle mass, strength, and function due to diminished mitochondrial oxidative capacity, heightened oxidative stress, and inflammatory cytokine-mediated proteolysis [12]. Accumulating evidence has revealed that aged and high-fat diet (HFD)-induced obese mice exhibit reduced indices evaluating skeletal muscle strength and function using grip strength and the rotarod test [13,14,15]. Mitochondrial oxidative capacity, regulated by the transcription of genes encoding enzymes involved in the tricarboxylic acid (TCA) cycle, fatty acid oxidation, oxidative phosphorylation (OXPHOS), and mitochondrial biogenesis, is intricately associated with muscle strength and function [16,17,18]. PPARGC1A (PGC1α) has been reported to enhance mitochondrial-oxidative-capacity-associated metabolism, including fatty acid oxidation, OXPHOS, mitochondrial biogenesis, and thermogenesis in skeletal muscle and brown adipose tissue via transcriptional activation of peroxisome proliferator-activated receptors (PPARs), estrogen-related receptors (ERRs), and nuclear respiratory factors (NRFs). Studies have demonstrated that NRFs, upregulated in response to endurance exercise, act as transcriptional activators of nuclear genes encoding numerous respiratory chain subunits in skeletal muscle, thereby improving mitochondrial oxidative capacity [19,20,21]. Studies on the gain-of-function and loss-of-function of PPARs and ERRs in skeletal muscle have revealed that these factors are essential for mitochondrial oxidative capacity in terms of energy production and adaptation to endurance exercise [18,22]. In comparison with type II glycolytic fibers, which contain fewer mitochondria and rely on glycolysis for energy production, increased PPARs are predominantly observed in type I oxidative fibers enriched in mitochondria and powered by the oxidation of glucose and fatty acids [23,24]. Excessive exercise induces an inflammatory response caused by oxidative stress, thereby delaying the recovery of damaged muscle fibers [25]. PPARγ has been reported to exhibit protective effects against cellular damage from oxidative stress by transcribing genes encoding antioxidant enzymes such as manganese-superoxide dismutase, catalase, glutathione peroxidase 3 (GPX3), and heme oxygenase 1 in skeletal muscle [26]. 

In this study, we successfully produced emodin and resveratrol-enriched fermented Pc (McPc) and assessed its improvement effect against nerve damage or HFD-induced skeletal muscle weakness in vivo. Mechanistically, McPc increased transcriptional regulators such as PPARγ and ERRγ related to mitochondrial biogenesis, OXPHOS, and antioxidants. McPc significantly enhanced skeletal muscle strength, motor coordination, and traction force in mice. These results strongly suggest that McPc can be applicable as a functional ingredient in functional and medicinal foods for strengthening skeletal muscle.

## 2. Materials and Methods

### 2.1. Cell Culture and Reagents

The C2C12 myoblasts were purchased from the American Type Culture Collection (ATCC; Rockville, MD, USA) and grown in Dulbecco’s modified Eagle’s medium (DMEM), supplemented with 10% fetal bovine serum (FBS) and 1% penicillin/streptomycin solution, in a humidified atmosphere of 95% air and 5% CO_2_ at 37 °C. At 85–95% confluence, myoblasts were induced to differentiate in DMEM using 2% horse serum (HS) once every day for an additional 5 days. The C2C12 myotubes were then treated with or without different concentrations of McPc (100 and 300 μg/mL), emodin (20 μM) or resveratrol (50 μM) for 24 h. Emodin and resveratrol were dissolved in DMSO. Dried stems of *Polygonum cuspidatum* (Pc) grown domestically were purchased and used to obtain main compounds. All chemical standards used in this study were purchased from Sigma-Aldrich (St. Louis, MO, USA) HPLC grade methanol, acetonitrile, and formic acid were purchased from Fisher Scientific (Fair Lawn, MA, USA). Ultrapure water was produced with a Milli-Q system.

### 2.2. Sample Extraction and Fermentation

Dried Pc was ground into powder using an electronic grinder. Briefly, 100 g of dried plant tissue was weight and 2000 mL of purified water was added. The extract was then extracted at 100 °C for 2 h and stopped overnight at room temperature. After the extraction process was completed, the supernatant was filtered and concentrated under vacuum. The concentrated sample was freeze-dried and stored at −80 °C before being used for analysis. The filtered Pc extract was inoculated with *Lactobacillus* spp. and fermented at 28 °C for 288 h. The culture medium was evaporated, lyophilized, −80 °C stored, and used for UPLC-QTOF/MS and HPLC analysis.

### 2.3. UPLC–QTOF/MS Experiments and Data Analysis

All Pc and McPc samples were milled into powders. Individual portions of the powdered samples (0.1 g) were dissolved in 60% water in methanol; the sample concentration was 1000 ppm. Unknown sample analysis was performed using a Waters ACQUITY UPLC I-Class system coupled with a Xevo-QTOF ESI mass spectrometer (Waters Corporation, Milford, MA, USA). UPLC separation was performed using a Waters ACQUITY reversed-phase C18 column (Waters ACQUITY UPLC^®^ BEH C18, Waters Corporation, Milford, MA, USA, 100 mm × 2.1 mm, 1.7 µm). Distilled water supplemented with 0.1% formic acid (A) and acetonitrile supplemented with 0.1% formic acid (B) were used as mobile phases at a flow rate of 0.4 mL/min. The gradient was optimized as follows: 0–12 min, 60% B; 12–12.1 min, 60 to 100% B; 12.1–15.3 min, 100% B; 15.3–15.4 min, 100 to 1% B; and 15.4–18 min, 1% B. In all cases, the injected volume was 1 μL, and the column temperature was maintained at 35 °C. The detection wavelengths of PDA were set at 210 nm, 254 nm, 280 nm, 360 nm, and max plot. A Waters AQUITY high-resolution Q-TOF mass spectrometer (Waters Corporation, Milford, MA, USA) was used to collect MS data in negative ion scan mode. The parameters were as follows: mass range: 100–1500 *m*/*z*; dry gas temperature: 350 °C; source temperature: 110 °C; dry gas flow rate: 800 L/hour; nebulizer: 35 psi; capillary voltage: 3000 V; con voltage: 40 V; and full MS scan at a resolution of 20,000. An MS2 scan was carried out using the data-dependent mode with a collision energy of 25–50 V. All MS data were collected and processed using a UNIFI 1.8 (Waters Corp.). Data within the UNIFI 1.8 were passed through the apex peak detection and alignment processing algorithms. The intensity of each ion was normalized to the total ion count to generate a data matrix with an *m*/*z* value, RT, and normalized peak area. The charged species, salt adducts, and fragments were all automatically aligned and grouped. The normalized peak areas of aligned compounds were exported to SIMCA-P+ 12.0 software (Umetrics, Umeå, Sweden) for PCA, PLS–DA, and OPLS–DA. These methods were constructed and optimized in previous studies [27,28].

### 2.4. HPLC Experiments and Data Analysis

HPLC-based quantitation of anthraquinone and stilbene of Pc is performed according to a previous report [29]. For the quantitative anthraquinones of Pc, all of the extracted samples were milled into powders, and individual portions of the powdered samples (0.2 g) were dissolved in methanol. The resulting samples were then extracted for 15 min using an ultrasonicator in a water bath (30 °C). The extracts were filtered using a GHP syringe filter (FALL Life Sciences, East Hills, NY, USA) and the elution was injected directly into the HPLC system for analysis. Emodin and emodin glucopyranosides were purchased from Sigma-Aldrich (St. Louis, MO, USA). Methanol, water (HPLC-grade) and formic acid (95%) were obtained from Fisher Scientific (Fair Lawn, MA, USA). Double-distilled water was obtained using a Millipore Milli-Q Plus water treatment system (Millipore, Bedford, MA, USA). The stock solutions of five standards were made at a concentration of 11.3 mg in a final volume of 100 mL of methanol. Working solutions of mixed standards at the concentrations of 7.1, 14.1, 28.3, 56.5 and 113 μg were made by dilution of stock solution in volumetric flasks with the mobile phase. Then, the standards were injected into the HPLC. High-performance liquid chromatography 20 μL samples were analyzed on a Zorbax eclipse XDB-C18 column (4.6 × 250 mm, 5 μm, Agilent, Santa Clara, CA, USA), which was maintained at 40 °C using an Agilent Infinity-1260 HPLC system. The mobile phases consisted of (A) water containing 0.1% (*w*/*w*) formic acid and (B) methanol. The HPLC elution conditions were optimized as follows: linear gradient from 20 to 35% B (0 to 13 min), 35 to 100% B (13 to 30 min), and 100 to 20% B (30 to 40 min), where it was held for 3 min. The flow rate was set at 1.0 mL/min, and the column and autosampler were maintained at 40 and 25 °C, respectively. The scan range for DAD detector system was set at 190 to 400 nm. Analogue output channel A was set at wavelength 254 nm with a bandwidth of 4 nm. In addition, for the quantification of stilbene, resveratrol and polydatin were purchased from Sigma-Aldrich (St. Louis, MO, USA). Acetonitrile (HPLC-grade) and acetic acid (99%) were obtained from Fisher Scientific (Fair Lawn, MA, USA). The stock solutions of five standards were made at a concentration of 11.2 mg in a final volume of 100 mL of methanol. Working solutions of mixed standards at concentrations of 3.5, 7, 14, 28, 56 and 112 μg were made by dilution of stock solution in volumetric flasks with the mobile phase. The mobile phases consisted of (A) water containing 0.1% (*w*/*w*) acetic acid and (B) acetonitrile. The HPLC elution conditions were optimized as follows: isocratic state to 26% B (0 to 20 min) maintained, linear gradient from 26 to 95% B (20 to 25 min) and 95 to 26% B (25 to 35 min), where it was held for 5 min. Analogue output channel A was set at wavelength.

### 2.5. RNA Seq and Quantitative Real-Time PCR (qRT-PCR)

TRIzol (Invitrogen, Carlsbad, CA, USA) and isopropanol (Sigma-Aldrich, St. Louis, MO, USA) were used for total RNA extraction in cultured mouse C2C12 cells and gastrocnemius muscle of mice, as described previously [6]. Gastrocnemius muscle was frozen with liquid nitrogen and frozen tissues were pulverized by using dry ice and liquid nitrogen. To extract total RNA, 1 mg samples of pulverized frozen tissues were homogenized with 1 mL of TRIzol using a bead homogenizer (Benchmark Scientific, Sayreville, NJ, USA). RNA purity was measured using a spectrophotometer (BioTek, Winooski, VT, USA) at 260 and 280 nm, and a ratio of ~1.8 is accepted for qRT-PCR. The reverse transcriptase and cDNA synthesis kit (Applied Biosystems, Foster City, CA, USA) and SYBR Green qPCR mixture (Applied Biosystems, Foster City, CA, USA) were as used for cDNA synthesis and qRT-PCR, respectively. Experimental Ct values were normalized and calculated to mouse β-actin gene. Details of the primer sequences, amplicon size, and gene accession number for qRT-PCR are described in Table 1 and Supplementary Table S1. RNA-Seq library construction was performed using TruSeq Stranded mRNA LT Sample Prep Kit (Illumina, Inc., Hayward, CA, USA) and sequenced following NovaSeq6000 System (Illumina, Inc., Hayward, CA, USA) User Guide at Macrogen (Seoul, Republic of Korea). Up- or down-regulated differentially expressed genes (DEGs) are listed in Supplementary Table S2.

### 2.6. Immunoblotting

The crude protein samples were extracted from cultured cells using protein extraction buffer (1% IGEPAL, 150 mM NaCl, 50 mM Tris-HCl (pH 7.9), 10 mM NaF, 0.1 mM EDTA, and a protease inhibitor cocktail) and frozen tissue sample were lysed in ice-cold RIPA buffer, containing a phosphatase inhibitor cocktail (Quartett, Berlin, Germany), lysates were centrifuged at 12,000× *g* for 20 min at 4 °C. Protein concentrations of the lysates were then quantified using the protein assay solution (BioRad, Hercules, CA, USA). Next, 30 μg of protein was separated using SDS-polyacrylamide gel electrophoresis (PAGE) and transferred onto a nitrocellulose membrane. To reduce non-specific reactions between primary antibodies and proteins, the membrane was reacted with 5% skim milk for 1 h at room temperature, after which it was immunoblotted with primary antibodies against myosin heavy chain (MyHC), LPL, PPARγ, OXPHOS, p-AMPK, p-mTOR, and p-ACC, as well as α-Tubulin, as an internal control, overnight at 4 °C. Antibody information is described in Supplementary Table S3. The membranes reacted with primary antibodies were washed three times with 1× tris-buffered saline (pH 7.4) containing 0.1% tween-20 (TBST) buffer, and then additionally reacted with horseradish peroxidase (HRP)-conjugated anti-mouse or anti-rabbit IgG. Target protein expression was visualized using ECL™ Western blotting detection reagent (GE Healthcare, Pittsburgh, PA, USA). The procedures followed for immunoblotting were as described previously [30].

### 2.7. Animal Experiment

For unilateral sciatic neurectomy (NTX), the animal experiments were approved and performed in accordance with the guidelines of the WoojungBio Inc. (Hwaseong, Republic of Korea) Institutional Animal Care and Committee (WJ2202045). Five-week-old ICR mice (*n* = 5) were purchased from Koatech (Animal Inc., Seoul, Republic of Korea). After acclimatization, unilateral sciatic neurectomy (NTX) was performed to induce hindlimb muscle atrophy according to the previously established method [31,32]. Briefly, all of the mice were anesthetized with 3% isoflurane in the mixture of 70% N_2_O and 28.5% O_2_, and anesthesia was maintained with 1 to 1.5% isoflurane in the mixture of 70% N_2_O and 28.5% O_2_. Right sciatic neurectomy was performed by removing a 1.5 cm section of the nerve in the mid-thigh, and a sham operation (nerve identification) was performed in the case of the sham groups as follows: animals were randomly divided into 3 groups of 5 mice each at 2 weeks after NTX treatment; sham control, NTX control and McPc-treated groups (75 mg/kg) based on the body weights (38.46 ± 2.05 g in the vehicle sham control group; 37.39 ± 2.00 g in the NTX group) and calf thicknesses (4.31 ± 0.21 mm in the vehicle sham control group; 2.84 ± 0.09 mm in the NTX group). Prepared and stored McPc stock solution (300 mg/kg in D.W.) was 4-fold diluted as 75 mg/mL with distilled water and then orally administered in a volume of 10 mL/kg of body weight as matched as 75 mg/kg of body weight, by oral gavages, once a day for 28 days from 2 weeks after NTX, respectively. An equal volume of distilled water was administered orally in sham and NTX control mice, instead of McPc. After performing gastrocnemius muscle thickness measurement at sacrifice, the gastrocnemius muscle masses were carefully separated from the tibia and fibula bones. Then, weights of individual induced gastrocnemius muscle masses were measured at g levels (absolute wet weights) using an automatic electronic balance and, to reduce the differences due to individual body weights, relative weights (% of body weights) were also calculated using body weight at sacrifice and absolute weight as follows: At 24 h before the last 28th administration of vehicle, NTX or NTX-McPc, grip strengths and rotarod test of individual mice were measured as muscular strengths using a computerized testing machine (RSIC software v 3.48, BIOSEB, Pinellas Park, FL, USA and X-PAD 3.0.2.6, UGO BASILE, Gemonio, VA, Italy). Briefly, grip strength was measured by holding the mouse’s tail and having the mouse hold the grid with its front paws, and then having a person pull the mouse’s tail with a certain force. In the Rotarod test, the mouse is placed facing the tester in the opposite direction of the rotating trade mill, the speed is gradually increased (4 to 40 rpm), and the time until the mouse loses balance and falls to the floor is measured. A sensor was placed on the floor to measure the time (s) it took to fall. In the rotarod test, the mouse was placed facing away from the tester in the opposite direction of the rotating trade mill, and the speed was gradually increased (4–40 rpm) to measure the time (s) until the mouse lost its balance and fell to the floor. For the high-fat diet (HFD) model, the animal experiments were approved and performed in accordance with the guidelines of the Konkuk University Institutional Animal Care and Committee (KU23133). Four-week-old C57BL/6J mice (*n* = 7) were purchased from Central Lab (Animal Inc., Seoul, Republic of Korea). After acclimatization, each candidate was fed freely in the normal chow diet (NCD) or HFD and replaced with new feed twice a week on the feed measurement day. The HFD experimental procedure was as described in previous reports [14,15]. The composition of the administration group and the manufacturing method of the formulated feed are shown in Table 2. After completion of the feeding experiment, blood and tissue samples were collected. Serum samples were collected entirely from the heart to obtain a large amount of mouse whole blood. After anesthetizing with Isoflurane 2% using a respiratory anesthetic machine, the skin in the center of the chest was incised and blood was collected from the heart of the mouse. The collected blood was transferred to a micro-SST (Serum-separating tube) and centrifuged at 3000 rpm for 10 min. For tissue samples, whole liver was removed and weighed. The middle-liver tissue was fixed in 10% NBF (neutral buffered formalin) and the left-liver tissue and the remaining tissue were stored frozen. The gastrocnemius muscle and inguinal WAT were extracted from both the left and right sides, weighed, stored frozen, and used for analysis. In addition, the separated tissues were pretreated to produce paraffin blocks and H&E-stained slides, and the separated blood was subjected to serum biochemical analysis. General symptoms such as appearance, behavior, and excrement were observed once daily during the observation period, and dead animals were identified. Body weight and feed were measured and recorded three times a week from the date of group separation and administration. In addition, to confirm efficacy after short-term administration, 100 mg/kg of each candidate was administered orally to another group of mice adapted to the substance once a day for 7 days. Compared to the control group, creatine was dissolved in distilled water at a concentration of 200 mg/kg and then orally administered during the same period as the McPc administration group. Twenty-four hours before the final seventh administration of candidate sample, muscle strength and mobility were measured using a computerized testing machine (RSIC software v 3.48, BIOSEB, Pinellas Park, FL, USA and X-PAD 3.0.2.6, UGO BASILE, Gemonio, VA, Italy) in the same manner as the NTX model.

### 2.8. Blood Biochemistry

To obtain serum for blood biochemistry, blood samples contained in a separation tube on the day of autopsy were centrifuged at 3000 rpm for 10 min. Serum liver function, lipid metabolism, and energy metabolism values were measured using an automatic analyzer commissioned by CM BioPATH (CRO, Hwasung, Republic of Korea).

### 2.9. Measurement of ROS

To determine oxidative stress, intracellular ROS were assessed using a DCF-DA fluorescence assay [33]. Cells were grown in black-well clear-bottom 96-well plates for 24 h. After washing with phosphate buffered saline (PBS) two times, cells were stained with 25 μM of DCF-DA for 30 min in the dark. Then, the cells were treated with various concentrations (50–200 μg/mL) of McPc in the presence or absence of H_2_O_2_ (500 μM) for 3 h. The fluorescence was determined at 485 (excitation)/535 (emission) nm using a microplate reader (Molecular Devices, Sunnyvale, CA, USA).

### 2.10. Statistical Analysis

Illustrative figures were represented using GraphPad Prism version 5 (GraphPad Software Inc., San Diego, CA, USA). Statistical analysis was performed using Student’s *t*-test for two experimental comparisons and one-way ANOVA Tukey’s post hoc test for multiple comparisons. A *p* value < 0.05 was considered statistically significant.

## 3. Results

### 3.1. Production of Emodin and Resveratrol-Enriched Polygonum cuspidatum (McPc) through Fermentation

**Figure 1:**
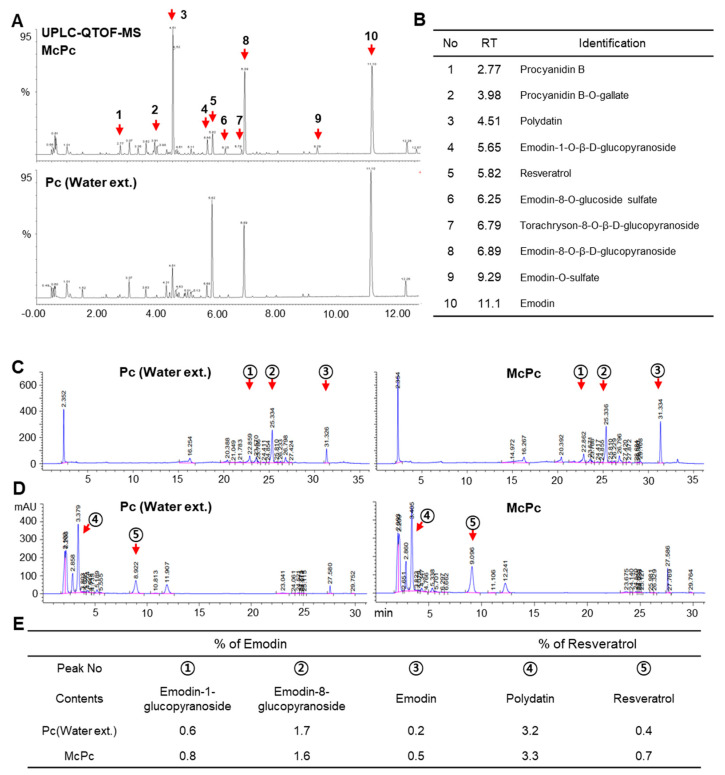
Qualification and quantification of primary compounds in the *Polygonum cuspidatum* (Pc) water extract and microbe complex-fermented *Polygonum cuspidatum* (McPc). (**A**) Ultra-high performance liquid chromatogram of McPc and Pc. (**B**) Identification of 10 compounds in Pc and McPc. (**C**) High-performance liquid chromatogram (HPLC) for quantitative anthraquinones in Pc and McPc. (**D**) HPLC for quantitative stilbenes in Pc and McPc. (**E**) Total anthraquinone and stilbene contents of the primary compounds in the stem of Pc.

Microorganism-based fermentation is widely employed to enhance the bioavailability and alter the composition of biologically active compounds in natural plant products [7]. *Lactobacillus* is a microorganism that is widely used in the fermented food industry [34]. As the capability of *Lactobacillus* in the synthesis and metabolism of vitamins is known to extend the bioavailability and production of several vitamins, including vitamin B12, vitamin C, and riboflavin (B2) [35], we attempted to produce fermented Pc (McPc) using *Lactobacillus* spp. Given that microorganism-based fermentation can increase the composition of functional ingredients, we assessed the changes in the main ingredients induced by Pc fermentation. Surprisingly, 10 major peaks showing alterations between McPc and Pc water extract were observed; these were analyzed using ultra-high-performance liquid chromatography–quadrupole time-of-flight mass spectrometry (UPLC–QTOF/MS). Figure 1A,B illustrate a total of 10 compounds identified through a comparison of retention times and accurate mass measurements with reference or library data. These compounds were divided into two categories based on their structural properties: anthraquinones and stilbenes. Subsequently, we validated the HPLC quantitative results for the five crucial compounds, revealing differences in peak intensity and area of anthraquinones and stilbenes between Pc and McPc. When measured using the standard curve, it was confirmed that the concentrations of essential compounds were altered. Notably, the concentrations of emodin and trans-resveratrol increased by up to 150% and 75%, respectively, through the fermentation process compared to the extract (Figure 1C–E). These findings suggest that McPc contains higher concentrations of emodin and resveratrol than Pc.

### 3.2. Identification of Target Genes Responsive to McPc

**Figure 2:**
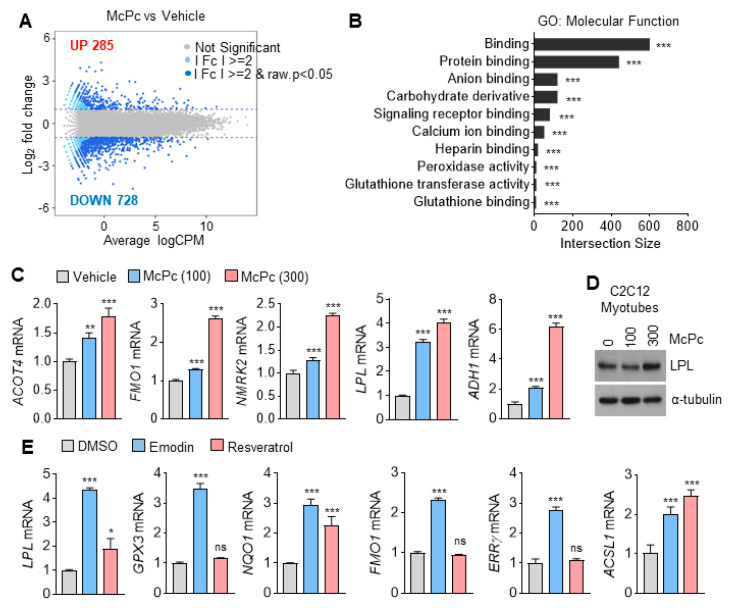
Transcriptome analysis in McPc-treated C2C12 myotubes. (**A**) MA plot (log2 fold change vs. average logCPM) of vehicle and McPc. (**B**) Gene ontology (GO) classification of differentially expressed genes. The graph shows approximately 10 GO terms with most genes annotated. The top 10 GO terms were assigned to the differentially expressed genes in C2C12 myotubes treated with or without McPc. (**C**) Gene expression of C2C12 myotubes incubated with McPc (μg/mL) for 24 h. (**D**) The protein level of LPL in C2C12 myotubes treated with various concentrations of McPc for 24 h. (**E**) Gene expression of C2C12 myotubes incubated with 20 μM emodin or 50 μM resveratrol for 24 h. The values represent the mean ± SD (*n* = 3); * *p* < 0.05, ** *p* < 0.01 and *** *p* < 0.001. ns = not significant. One-way analysis of variance (ANOVA) Tukey’s post hoc test was performed for statistical analysis.

Resveratrol has been reported to enhance muscle activity and energy expenditure in HFD mice by regulating PGC1α (PPARGC1A) acetylation-mediated metabolic gene expression [14,15]. Emodin, known for various health benefits such as anti-inflammatory, anticancer, antiviral, antifibrosis, anticardiovascular disease, and antioxidant activity [36,37], was investigated for its impact on the transcriptome in McPc-treated C2C12 myotubes. A total of 285 upregulated and 728 downregulated genes were observed in McPc-treated myotubes (Figure 2A). Additionally, molecular functions such as protein binding, signaling receptor binding, and glutathione-transferase-activity-associated gene sets were identified as responsible target genes of McPc (Figure 2B). Concordant with RNA-sequencing data, increased mRNA expression of lipoprotein lipase (LPL), flavin-containing monooxygenase 1, nicotinamide riboside kinase 2, acyl-coenzyme A thioesterase 4, and alcohol dehydrogenase 1, as well as elevated LPL protein levels, were observed in McPc-treated C2C12 myotubes (Figure 2C,D). To determine which active ingredient within McPc affects the alteration of gene expression, C2C12 myotubes were exposed to emodin or resveratrol (Figure 2E). These results suggest that emodin and resveratrol-enriched McPc may increase antioxidant-, lipid-metabolism-, and energy-expenditure-associated gene expression in cultured C2C12 myotubes. 

### 3.3. McPc Prevents Oxidative Stress In Vitro and In Vivo

**Figure 3:**
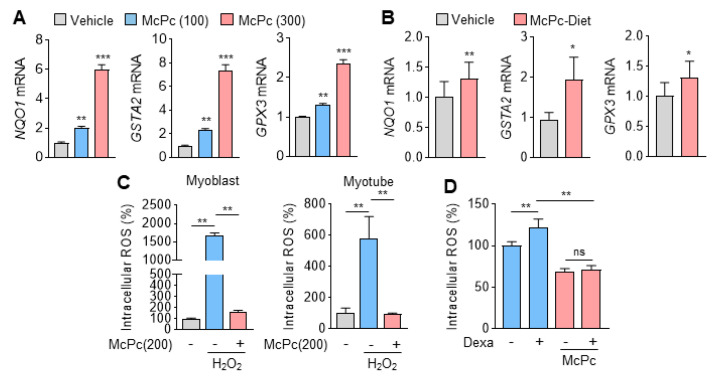
Antioxidant activity of fermented McPc in vitro and in vivo. (**A**,**B**) Antioxidant-related genes, NQO1, GSTA2, and GPX3, mRNA expression of C2C12 myotubes treated with McPc (*n* = 3) and McPc-diet mice (*n* = 5). (**C**) Intracellular reactive oxygen species (ROS) levels of C2C12 myoblasts and myotubes (*n* = 4). C2C12 cells were treated with 200 μg/mL of McPc in presence or absence of H_2_O_2_ (100 μM). (**D**) Intracellular ROS levels of 100 μg/mL McPc-treated C2C12 myotubes incubated with dexamethasone (100 μM) to induce ROS (*n* = 4). The values represent the mean ± SD; * *p* < 0.05, ** *p* < 0.01, and *** *p* < 0.001. ns = not significant. One-way ANOVA Tukey’s post hoc test was conducted for multiple comparisons, and the two-tailed unpaired Student’s *t*-test was conducted for single comparisons.

In accordance with transcriptome analysis, Figure 3A demonstrates a significant increase in genes that encode detoxification and antioxidant enzymes in McPc-treated C2C12 myotubes. Furthermore, elevated mRNA expression of NAD(P)H: quinone oxidoreductase 1 (NQO1), glutathione S-transferase alpha 2 (GSTA2), and GPX3 were observed in the gastrocnemius muscle of mice that were administered McPc. Intracellular reactive oxygen species (ROS) in hydrogen peroxide (H_2_O_2_)-treated C2C12 myoblasts and myotubes were significantly reduced by McPc treatment. Given that cortisol, a stress hormone, is known to cause oxidative stress-associated chronic diseases, including skeletal muscle atrophy [38], we investigated whether McPc can prevent dexamethasone (as a cortisol analog)-induced oxidative stress in C2C12 myotubes. McPc effectively prevented dexamethasone-induced intracellular ROS in C2C12 myotubes (Figure 3D), indicating that emodin and resveratrol-enriched McPc may exert protective effects against oxidative stress-associated aging and chronic diseases. 

### 3.4. McPc Enhances Expression of Mitochondrial Oxidative Capacity-Associated Genes in Cultured Myotubes

**Figure 4:**
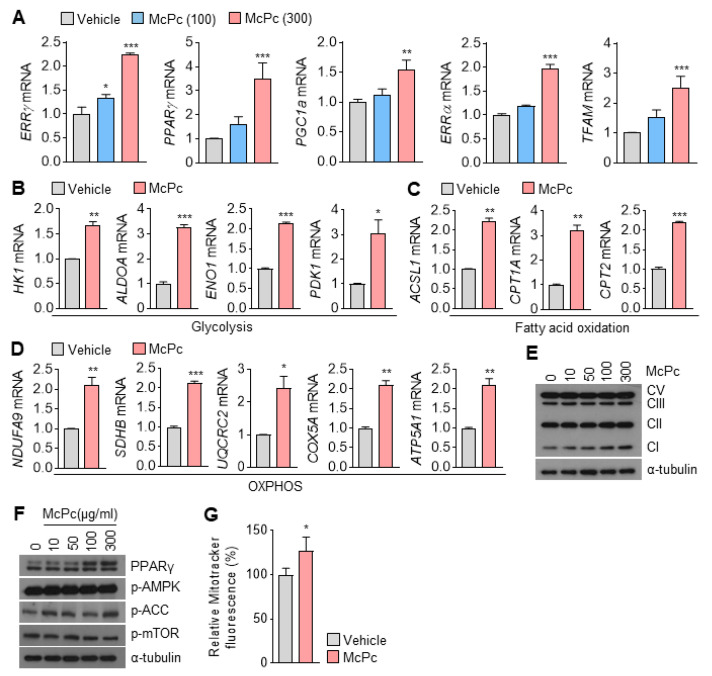
Expression of genes associated with mitochondrial oxidative capacity in McPc-treated C2C12 myotubes. (**A**) Expression of mRNA-encoding transcription factors and coactivators related to mitochondrial oxidative capacity in C2C12 myotubes incubated with McPc for 24 h. (**B**) Glycolysis-related gene expression. (**C**) Fatty-acid-oxidation-related gene expression. (**D**) Oxidative phosphorylation (OXPHOS)-associated gene expression. (**E**) Levels of OXPHOS complex proteins (CI: NDUFB8, CII: SDHB, CIII: UQCRC2, CV: ATP5A) in C2C12 myotubes treated with McPc for 24 h. (**F**) Protein levels in C2C12 myotubes treated with McPc for 24 h. (**G**) Mitochondrial content of 100 μg/mL McPc-treated C2C12 myotubes measured using the MitoTracker staining assay. The density of MitoTracker Deep Red FM fluorescent was evaluated using a microplate reader (λex 644 nm and λem 665 nm). The values represent mean ± SD; * *p* < 0.05, ** *p* < 0.01, and *** *p* < 0.001. One-way ANOVA Tukey’s post hoc test was conducted for multiple comparisons and two-tailed unpaired Student’s *t*-test was conducted for single comparisons.

PPARs and ERRs, acting as nuclear receptors, play pivotal roles as transcriptional factors in the regulation of mitochondrial oxidative capacity, encompassing OXPHOS, fatty acid oxidation, the TCA cycle, and mitochondrial biogenesis in skeletal muscle [18]. Our RNA-sequencing data demonstrate a significant increase in ERRγ mRNA in McPc-treated C2C12 myotubes. Consistent with the RNA-sequencing results, genes encoding transcription factors such as ERRγ, PPARγ, ERRα, PGC1α, and mitochondrial transcription factor A (TFAM), which promote mitochondrial oxidative capacity, were upregulated in a dose-dependent manner in McPc-treated C2C12 myotubes (Figure 4A). Given that these transcription factors regulate energy metabolism through gene expression, we further investigated the expression of energy metabolism-associated genes. McPc treatment led to increased expression of glycolysis-related genes (HK1, ALDOA, ENO1, and PDK1) in C2C12 myotubes (Figure 4B). Furthermore, McPc increased the expression of fatty acid oxidation (Figure 4C) and OXPHOS (Figure 4D)-associated genes in C2C12 myotubes. Consistently, a dose-dependent increase in the protein levels of mitochondrial OXPHOS complex I (NDUFB8) was observed in McPc-treated C2C12 myotubes (Figure 4E). Additionally, PPARγ protein levels were elevated in McPc-treated C2C12 myotubes (Figure 4F). Notably, there were no significant effects of McPc on AMP-activated protein kinase signaling, a regulator of mitochondrial oxidative capacity, observed between McPc-treated and non-treated C2C12 myotubes in skeletal muscle (Figure 4F). In line with the observed increase in mitochondrial-oxidative-capacity-related transcription factors such as TFAM and PGC1α, the enhanced mitochondrial content was verified via the difference in MitoTracker fluorescence in McPc-treated C2C12 myotubes compared to non-treated C2C12 myotubes (Figure 4G). These findings highlight that McPc can stimulate the expression of genes related to mitochondrial oxidative capacity, including components of the respiratory apparatus and oxidative enzymes, in skeletal muscle.

### 3.5. McPc Improves Skeletal Muscle Strength and Motor Function

**Figure 5:**
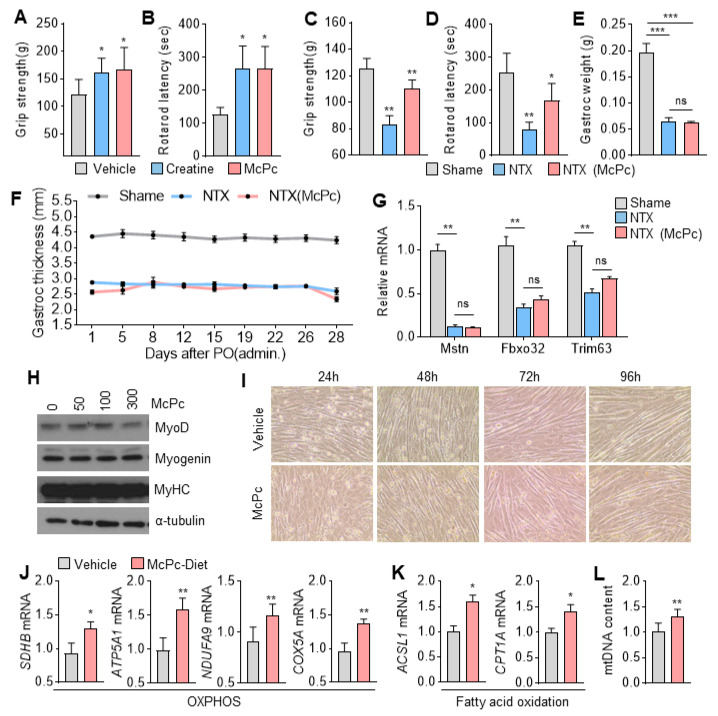
Skeletal muscle strength and function in McPc-treated mice. (**A**) Grip strength and (**B**) rotarod latency in McPc-treated mice. McPc (100 mg/kg) was orally administered once a day for 7 days (*n* = 5). (**C**–**F**) Effect of McPc treatment on (**C**) grip strength, (**D**) rotarod latency, (**E**) gastrocnemius muscle weight, and (**F**) thickness in sciatic neurectomy (NTX) in mice (*n* = 5). McPc (75 mg/kg) was orally administered once a day for 28 days from 2 weeks after NTX. (**G**) The mRNA expression of Mstn, Fbxo32, and Trim63 in vehicle or McPc-treated NTX mice model. (**H**) Protein levels of muscle differentiation markers, MyoD, myogenin, and MyHC, in C2C12 myotubes incubated with McPc in a dose-dependent manner for 24 h. (**I**) Brightfield image by differentiation time of C2C12 myotubes treated with 100 μg/mL McPc in differentiation media (2% horse serum). (**J**) OXPHOS and (**K**) fatty-acid-oxidation-related gene expression in gastrocnemius muscle of vehicle or McPc-treated NTX mice. (**L**) Mitochondrial DNA content in gastrocnemius muscle of vehicle or McPc-treated NTX mice. The values represent the mean ± SEM; * *p* < 0.05, ** *p* < 0.01, and *** *p* < 0.001. ns = not significant. One-way ANOVA Tukey’s post hoc test was conducted for multiple comparisons and two-tailed unpaired Student’s *t*-test was conducted for single comparisons.

Given the association between mitochondrial oxidative capacity in skeletal muscle and muscle strength and motor function, we investigated whether McPc could improve these parameters. Surprisingly, mice administered with McPc exhibited increased muscle strength (Figure 5A) and significantly improved motor coordination and traction force (Figure 5B), as evidenced by the rotarod and grip strength tests, compared to non-treated mice. To elucidate the potential therapeutic benefits of McPc on skeletal muscle dysfunction, we assessed its effects on muscle strength in a sciatic neurectomy (NTX)-induced hindlimb muscle atrophy model in mice. McPc administration significantly rescued reduced muscle strength, motor coordination, and traction force in NTX mice (Figure 5C,D). Notably, there were no significant differences in gastrocnemius weight and thickness between McPc-treated NTX mice and non-treated NTX mice (Figure 5E,F), indicating that McPc may improve muscle strength and motor function independently of skeletal muscle fiber growth. The decreased expression of proteolysis-related genes, such as Mstn, Fbxo32, and Trim63, in the gastrocnemius muscle of NTX mice was not reversed by McPc treatment (Figure 5G). Additionally, the thickness of differentiated C2C12 myotubes, as well as the expression of muscle differentiation and development markers such as myoblast determination protein 1 (MyoD), myosin heavy chain (MyHC), and myogenin, remained unchanged in McPc-treated cells compared to non-treated cells (Figure 5H,I). Considering that McPc increases the expression of OXPHOS and fatty-acid-oxidation-related genes in cultured C2C12 myotubes, we measured the expression of OXPHOS and fatty acid oxidation-related genes in the gastrocnemius muscle isolated from McPc-administered mice. Consistent with the results of the muscle strength and motor function tests, OXPHOS (Figure 5J) and fatty acid oxidation (Figure 5K)-associated genes, as well as mitochondrial DNA content (Figure 5L), were significantly increased in the gastrocnemius muscle of McPc-treated mice compared to non-treated mice. These findings suggest that McPc may enhance OXPHOS and fatty acid oxidation, promoting energy expenditure through mitochondrial biogenesis. Overall, these results indicate that McPc exerts beneficial effects against skeletal muscle atrophy and weakness induced by motor nerve damage. 

### 3.6. McPc Improves HFD-Induced Impaired Skeletal Muscle Strength, Fatty Liver, Hyperglycemia, and Hyperlipidemia

**Figure 6:**
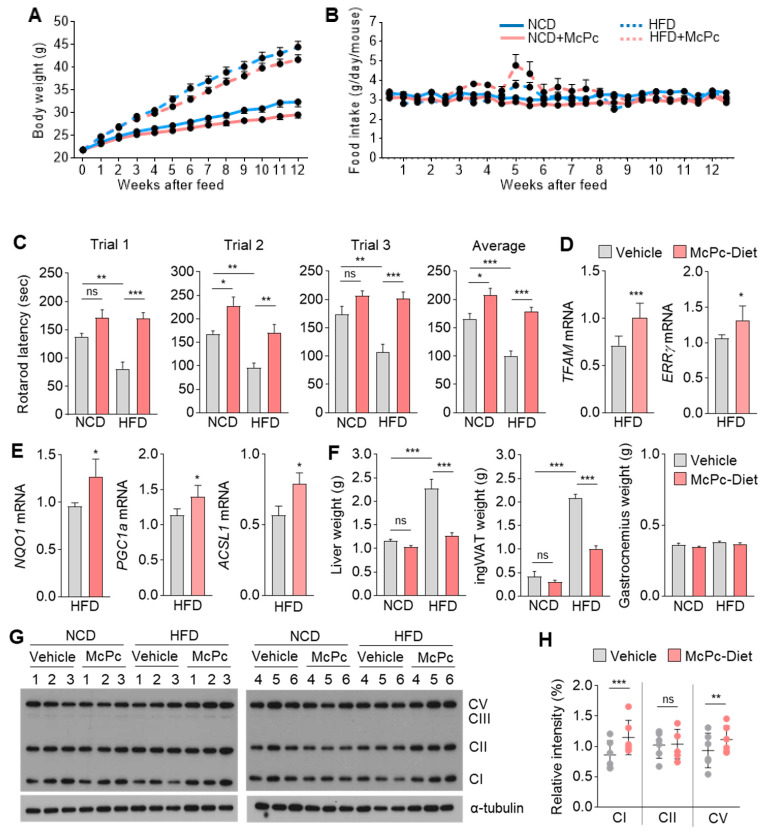
McPc rescues impaired skeletal muscle strength and function caused by HFD. (**A**) Body weight and (**B**) food intake of male C57BL/6 mice fed a normal chow diet (NCD) or an HFD supplemented with or without McPc over 12 weeks (*n* = 9). (**C**) Effect of McPc diet on rotarod latency in NCD and HFD mice. (**D**) TFAM and ERRγ mRNA expression in the gastrocnemius muscle of non-treated or McPc-treated HFD mice. (**E**) NQO1, PGC1α, and ACSL1 mRNA expression in the gastrocnemius muscle of non-treated or McPc-treated HFD mice. (**F**) Tissue weight of liver, inguinal white adipose (ingWAT), and gastrocnemius of NCD and HFD mice supplemented with vehicle or McPc. The values represent the mean ± SEM (*n* = 9); * *p* < 0.05, ** *p* < 0.01, and *** *p* < 0.001. One-way ANOVA Tukey’s post hoc test for multiple comparisons and two-tailed unpaired Student’s *t*-test for single comparisons were performed for statistical analysis. (**G**) Expression of OXPHOS complex proteins in the gastrocnemius muscle of NCD and HFD mice supplemented with vehicle or McPc (*n* = 6). (**H**) Densitometric analysis of Western blots revealing OXPHOS (complex I, II, and V) expression. Six independent Western blots were quantified with densitometry using Image J Software Version 1.53. The protein levels were normalized with respect to the corresponding intensities of α-tubulin. The values represent the mean ± SD (*n* = 6); ** *p* < 0.01 and *** *p* < 0.001. ns = not significant. Unpaired Student’s *t*-test was performed for statistical analysis.

Obesity and aging are widely recognized risk factors for diminishing skeletal muscle strength and motor function [11,39]. Therefore, we further investigated whether McPc could reverse the impaired muscle activity induced by obesity. Figure 6A demonstrates that McPc significantly reduces body weight gain in HFD challenged mice, and Figure 6B shows that the reduction in body weight by McPc administration is independent of differences in food intake. A repeated rotarod test revealed the remarkable rescue of reduced motor coordination and traction force in HFD mice by McPc treatment (Figure 6C). In addition to the favorable effects of McPc on skeletal muscle strength and motor function, McPc administration significantly reversed hyperglycemia in HFD mice (Supplementary Figure S1A,B). Interestingly, despite the improvement in hyperglycemia by McPc treatment, blood insulin levels remained unchanged between non-treated and McPc-treated HFD mice (Supplementary Figure S1C). Compared to non-treated HFD mice, McPc-treated HFD mice exhibited reduced fatty liver (Supplementary Figure S1D), lower levels of blood low-density lipoprotein and total blood cholesterol (Supplementary Figure S1E). The abnormally elevated blood alanine aminotransferase levels in HFD mice were reversed by McPc administration (Supplementary Figure S1F). These findings suggest that McPc exhibits protective effects against HFD-induced non-alcoholic fatty liver (NAFL), hyperglycemia, and hyperlipidemia. Increased mitochondria in the gastrocnemius muscle, as evidenced by upregulated TFAM and ERRγ mRNA expression, were observed in HFD-challenged mice (Figure 6D). Figure 6E reveals increased mRNA levels of NQO1 (antioxidant), PGC1α (mitochondrial biogenesis), and ACSL1 (fatty acid oxidation) in the gastrocnemius muscle of HFD-challenged mice. McPc-administered HFD mice exhibited reduced liver and inguinal white adipose (ingWAT) weights, but not gastrocnemius muscle weight, compared to only HFD mice (Figure 6F). Intriguingly, increased levels of mitochondrial OXPHOS complex I (NDUFB8) and complex III (UQCRC2) were observed in the gastrocnemius muscle of McPc-administered HFD mice (Figure 6G,H). These results indicate that McPc may activate mitochondrial oxidative capacity, thereby rescuing the impaired skeletal muscle function caused by obesity. 

## 4. Discussion

In this study, we produced fermented *Polygonum cuspidatum* extract (McPc) with *Lactobacillus* spp. UPLC–QTOF/MS and HPLC analysis showed that McPc contains higher concentrations of emodin and trans-resveratrol than Pc. Many reports have demonstrated that trans-resveratrol has health benefits against multiple types of chronic diseases, such as sarcopenia [40,41], diabetes [42], and cardiovascular diseases [43]. Interestingly, a recent study has revealed that cis-resveratrol has a protective effect against oxidative stress-induced neuronal damage, while trans-resveratrol causes neuronal toxicity in rat cortical neurons [44]. McPc-administered mice received McPc containing emodin and trans-resveratrol for over 12 weeks in an HFD animal experimental setting; grip strength and rotarod tests revealed that the McPc-administered mice displayed improved skeletal muscle strength and motor coordination. Given that neurodegeneration (such as Parkinson’s disease) caused by neuronal toxicity leads to decreased latency of the rotarod test [45], we speculate that McPc containing trans-resveratrol is not a cause of neuronal toxicity.

Mitochondrial oxidative capacity in skeletal muscle, including total mitochondrial content, surface area of mitochondrial inner membranes, and respiratory activities, plays a pivotal role in determining muscle function, fiber types, strength, endurance, and sensorimotor function [46]. Environmental stressors such as exercise, caloric restriction, low temperature, and oxidative stress influence mitochondrial biogenesis and energy metabolism, including OXPHOS, the TCA cycle, and fatty acid oxidation, regulated by transcriptional factors and coactivators such as PPARs, ERRs, NRFs, TFAM, and PGC1α [47].

In our study, McPc treatment resulted in the upregulation of PPARγ, PGC1α, ERRα, ERRγ, and TFAM in C2C12 myotubes and gastrocnemius muscle in mice compared to the control group. Furthermore, transcripts encoding enzymes involved in glycolysis, fatty acid oxidation, and OXPHOS, downstream targets of PPARs, PGC1α, and ERRs, exhibited increased expression in McPc-treated C2C12 myotubes and gastrocnemius muscle in mice. Increased mitochondrial respiratory complexes were observed in HFD-fed mice supplemented with McPc compared to those fed only HFD. Enhanced MitoTracker fluorescence and mitochondrial DNA copy number indicated increased mitochondrial content in vitro and in vivo with McPc treatment. Pc extract has been found to recover a poor mitochondrial structure and increase the levels of Na+-K+-ATPase, along with respiratory complexes I and II in rat liver [48]. In addition, Pc components including polydatin, aloe-emodin, emodin, and luteolin were detected in mitochondrial lysates driven from Pc-administered rat liver [48], suggesting that Pc components can directly regulate mitochondrial structure and function. Emodin, a single compound contained within Pc extract, has been found to enhance mitochondrial ATP generation capacity and antioxidant components in both male and female rat heart muscle [49]. Song et al. showed that emodin promotes glucose uptake and utilization by activating AMPK signaling in L6 myotubes, and consequently improves insulin sensitivity in HFD-induced diabetic mice [50]. Resveratrol promotes mitochondrial oxidative capacity and enhances skeletal muscle function and strength [14,15]. Consistent with these previous observations, we found that emodin and resveratrol-enriched McPc increased mitochondrial DNA, respiratory complexes I and V, fatty acid oxidation, and OXPHOS-related gene expression in gastrocnemius muscle. These findings suggest that emodin and resveratrol-enriched McPc induce mitochondrial biogenesis and the expression of genes associated with mitochondrial energy metabolism through the upregulation of key regulators. Given the close association between increased mitochondrial content and respiratory complexes with improved skeletal muscle strength and function, we propose that McPc may contribute to increased skeletal muscle strength and function. Indeed, mice administered McPc exhibited enhanced skeletal muscle strength, motor coordination, and traction force, as assessed by grip strength and rotarod tests. Moreover, these beneficial effects of McPc on skeletal muscle strength and function were confirmed in an NTX-induced hindlimb muscle atrophy model, suggesting that McPc can mitigate the weakness of skeletal muscle strength and function caused by nerve damage. 

In skeletal muscle, ROS can be produced as by-products of mitochondrial OXPHOS due to their high oxygen consumption [51]. Accumulating evidence has revealed that inappropriate concentrations of ROS in skeletal muscle affect muscle mass and reduce muscle strength. These phenomena can be attributed to increased proteolysis, decreased myogenesis, and disrupted mitochondrial oxidative capacity, which are associated with the pathophysiology of various muscle diseases, including dystrophy and atrophy [52]. In our gene expression profiling, we found that McPc-treated C2C12 myotubes exhibited increased expression of NRFs target downstream genes encoding antioxidants, such as GPX3, NAD(P)H: NQO1, and GSTA2. This antioxidant effect was confirmed in McPc-administered gastrocnemius muscle in mice using quantitative reverse transcriptase-polymerase chain reaction (qRT-PCR), indicating the role of McPc as an antioxidant in both in vitro and in vivo settings. Previous research has shown that Pc containing resveratrol attenuates oxidative stress and increases plasma levels of pro-inflammatory cytokines; healthy professional basketball players (*n* = 10) received 200 mg of Pc extract containing 40 mg of trans-resveratrol for 6 weeks, and significantly decreased plasma concentrations of TNF-α and IL-6 were observed compared with a placebo group (*n* = 10) [40]. In another study on humans, Pc extract containing trans-resveratrol decreased the plasma concentrations of TNF-α, IL-6, and C-reactive peptide in normal weight and age-matched healthy subjects [41]. Sarcopenic obesity, the loss of skeletal muscle mass and function associated with excessive adipose-induced inflammatory cytokines and oxidative stress, causes diminished quality of life and increased risk of mortality [39]. Accumulated evidence has shown that abnormally produced pro-inflammatory cytokines, such as TNF-α and IL-6, from adipocytes themselves or infiltrated macrophages within WAT reduce the mass of skeletal muscle and strength in both men and women with sarcopenic obesity [53,54,55]. In other cases, tumor cells driven by TNF-α and IL-6 directly affected wasting of skeletal muscle (a process that is also called cancer cachexia) [56,57]. In addition, we previously showed that Pc extract has a protective effect against wasting of skeletal muscle in a lung-cancer-cell-bearing mouse model [6]. Together, these observations provide new insight into how McPc decreases obesity-induced plasma concentrations of inflammatory cytokines such as TNF-α and IL-6 and provides the fundamental mechanism by which McPc containing emodin and trans-resveratrol has protective effect against weakening of skeletal muscle strength and function caused by abnormally increased inflammatory cytokines.

However, the therapeutic efficacy of McPc in improving the loss of gastrocnemius muscle mass in the NTX-induced hindlimb muscle atrophy model was not significant. McPc administration did not reverse the decreased expression of genes involved in proteolysis, such as myostatin (Mstn), F-box only protein 32 (Fbxo32, also known as atrogin-1), and muscle RING-finger protein-1 (Trim63, also known as MuRF1), in the gastrocnemius muscle of the NTX model. Additionally, no effects of McPc on C2C12 myoblast differentiation into myotubes were observed. Despite these findings, our results suggest that the improved skeletal muscle strength and function with McPc are partially associated with mitochondrial oxidative capacity, independently of proteolysis and myogenesis, through its antioxidant effects. In addition, we previously showed that Pc extract containing emodin significantly decreased the proteolysis of skeletal muscle in lung-cancer-cell-bearing mice [6]. However, NTX-induced proteolysis of skeletal muscle was not recovered by McPc administration. These results indicate that the protective effect of McPc against proteolysis of skeletal muscle depends on the major causative factor of wasting of skeletal muscle.

Mice fed an HFD were protected from the development of obesity, fatty liver, hyperglycemia, and hyperlipidemia when administered with McPc. Importantly, McPc supplementation rescued decreased skeletal muscle strength, motor coordination, and traction force in HFD-fed mice. Studies have highlighted the protective effects of Pc extracts and its bioactive components (resveratrol, polydatin, and emodin) against HFD-associated metabolic dysregulation, such as obesity, fatty liver, hyperglycemia, hyperlipidemia, and insulin resistance [58,59,60]. Resveratrol, a SIRT1 activator, has exhibited improved skeletal muscle function and strength, dependent on PGC1α-mediated mitochondrial oxidative capacity, in HFD-fed mice, extending lifespan and rescuing metabolic dysregulation [14,15]. In our study, McPc increased TFAM, mitochondrial DNA, and MitoTracker fluorescence intensity, indicative of enhanced mitochondrial biogenesis, in C2C12 myotubes and gastrocnemius muscle in mice. These findings suggest that McPc improves skeletal muscle function through ATP production, energy expenditure, and enhanced aerobic capacity. The observed protective effects of McPc against skeletal muscle weakness and function extend beyond the control of HFD-induced metabolic dysregulation, including obesity, NAFL, hyperglycemia, and hyperlipidemia.

## 5. Conclusions

Skeletal muscle weakness, sarcopenia, is closely associated with increased risk of mortality, and the development of functional ingredients for improving skeletal muscle function is necessary. In this study, we successfully developed emodin- and resveratrol-enriched Pc through fermentation with a microorganism complex containing *Lactobacillus* spp., referred to as McPc. Through preclinical studies, we observed that McPc enhances skeletal muscle strength, motor coordination, and traction force in mice. Taken together, our results demonstrate that emodin and resveratrol-enriched McPc is a valuable therapeutic and preventive ingredient in functional foods for recovering weakness of skeletal muscle.

## Figures and Tables

**Table 1:** Primer sequences for qRT-PCR.

Gene	Forward Sequences (5′-3′)	Reverse Sequences (5′-3′)
LPL	GCCCGAGGTTTCCACAAATA	GCTGAAGTAGGAGTCGCTTATC
GPX3	ATCCTGCCTTCTGTCCCTGCTC	TGGTGAGGGCTCCATACTCGTA
NQO1	GACAACGGTCCTTTCCAGAATA	CTCTGAATCGGCCAGAGAATG
ERRγ	CAGAAGTACAAGCGCAGAATAGA	CACCAACAAATGCGAGACAATC
ADH1	GAAGAAGTCTACAAGGACCCATC	CACCGCAGCTTTGCATTT
NMRK2	GGCCCATGTACCAGAAGTATAG	CCTCCAGAACTTGATGGAAGAG
ACOT4	GAAGAAGCAGTGCGGTACAT	GCCATGATCAGACAGACATCAG
GSTA2	ATACAGAGTCCGGAAGATTTGG	GGTGGCGATGTAGTTGAGAA
PPARγ	CAGGCTTCCACTATGGAGTTC	GGCAGTTAAGATCACACCTATCA
PGC1α	AAACTGACTTCGAGCTGTACTT	CCCATGAGGTATTGACCATCTC
ERRα	TGCTCAGCTCTCTACCCAAAC	GGACAGCTGTACTCGATGCTC
TFAM	GGAATGTGGAGCGTGCTAAAA	GCTGGAAAAACACTTCGGAATA
HK1	AGGGCGCATTACTCCAGAG	CCCTGTGGGTGTCTTGTGTG
ALDOA	CGTGTGAATCCCTGCATTGG	CAGCCCCTGGGTAGTTGTC
ENO1	TGCGTCCACTGGCATCTAC	CAGAGCAGGCGCAATAGTTTTA
PDK1	GGACTTCGGGTCAGTGAATGC	TCCTGAGAAGATTGTCGGGGA
ACSL1	GCTTGTGGATGTGGAAGAAATG	TCTTGCTGGGTCTTTCAAGTAG
CPT1A	CTCTGCTGCATGGTAGATGTT	GCTCTGCGTTTATGCCTATCT
CPT2	CCTGCTCGCTCAGGATAAACA	GTGTCTTCAGAAACCGCACTG
NDUFA9	GTCCGCTTTCGGGTTGTTAGA	CCTCCTTTCCCGTGAGGTA
SDHB	AATTTGCCATTTACCGATGGGA	AGCATCCAACACCATAGGTCC
UQCRC2	AAAGTTGCCCCGAAGGTTAAA	GAGCATAGTTTTCCAGAGAAGCA
COX5A	GCCGCTGTCTGTTCCATTC	GCATCAATGTCTGGCTTGTTGAA
ATP5A1	TCTCCATGCCTCTAACACTCG	CCAGGTCAACAGACGTGTCAG
Mstn	CAGGAGAAGATGGGCTGAATC	AGTGCTCATCGCAGTCAAG
Fbxo32	ACCCAAGAAGAGAGCAGTATG	GACTCCCAGCCATCCAATTA
Trim63	GGACTACTTTACTCTGGACTTAGAAC	CAGCCTCCTCTTCTGTAAACTC
β-actin	CCTAAGGCCAACCGTGAAA	TGGTACGACCAGAGGCATA

**Table 2:** Nutrients contents in NCD, HFD and contained McPc feeds.

Nutrition	NCD	NCD-McPc 4%	HFD	HFD-McPc 4%
Casein	200	189.93	200	191.36
Corn starch	397.486	368.33	72.8	47.78
Dextrose	132	132	-	-
Maltodextrin	-	-	100	100
Sucrose	100	100	172.8	172.8
Cellulose	50	50	50	50
Soybean Oil	70	69.23	25	24.34
t-Butylhydroquinone	0.014	0.014	-	-
Lard	-	-	177.5	177.5
Mineral mix	-	-	10	10
Dicalcium phosphate	-	-	13	13
Calcium carbonate	-	-	5.5	5.5
Potassium citrate	-	-	16.5	16.5
Salt Mix	35	35	-	-
Vitamin Mix	10	10	10	10
L-cystine	3	3	3	3
Choline Bitartrate	2.5	2.5	2	2
FD&C red dye	-	-	0.05	0.05
McPc	-	40	-	34.33
Total (g)	1000	1000	858.15	858.15

## Data Availability

All materials generated in this study are available from the corresponding author on reasonable request. Differentially Expressed Genes (DEGs) obtained from RNA-Sequencing is included in Supplementary Materials and is publicly available.

## Supplementary Materials

The following supporting information can be downloaded at: https://www.mdpi.com/article/10.3390/nu16020305/s1, Figure S1: McPc diminishes HFD-induced hyperglycemia, fatty liver, and hyperlipidemia; Table S1: The amplicon size and gene accession number for qRT-PCR, Table S2: List of differentially expressed genes (DEGs) obtained from RNA-Sequencing, Table S3: Antibody information. Original UPLC-QTOF-MS and HPLC chromatogram.
