# Supplementary material for: Effects of Fermented Polygonum cuspidatum on the Skeletal Muscle Functions
Source: Nutrients. 2024 Jan 19;16(2):305. doi: 10.3390/nu16020305 (PMC10818974; doi:10.3390/nu16020305)
Supplement: Supplementary file 1 [file nutrients-16-00305-s001.zip › Figure S1.docx]

**
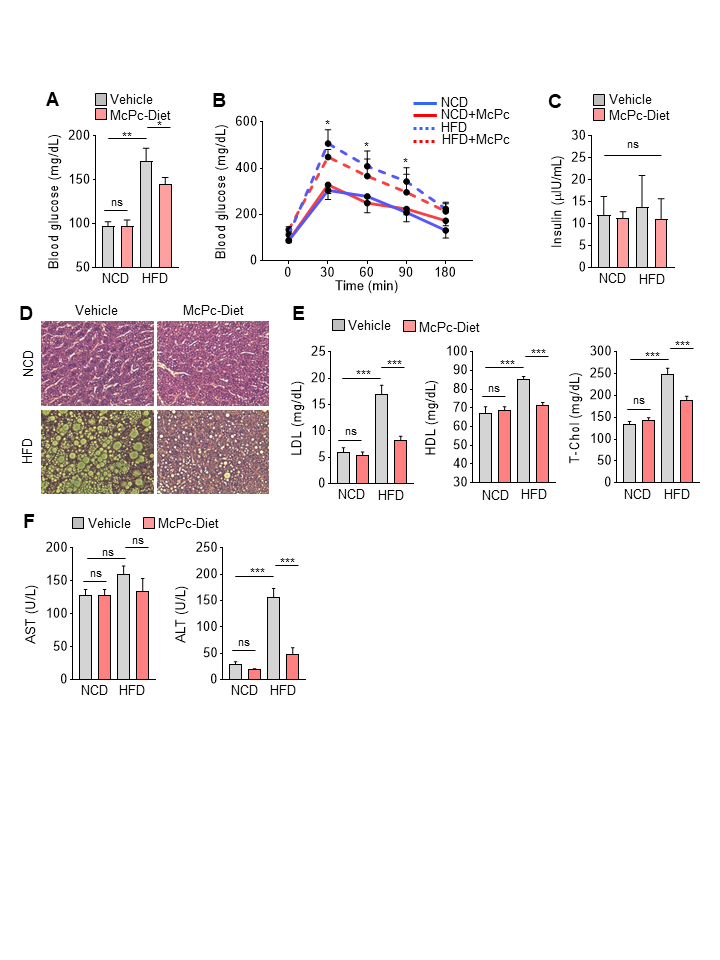
**

**Figure S1.** **McPc diminishes HFD-induced hyperglycemia, fatty liver and hyperlipidemia.** (A) Effect of McPc-diet on fasting blood glucose level, (B) glucose tolerance test (GTT) and (C) blood insulin level in NCD and HFD mice. (D) H&E staining of livers (magnification, 400x) from NCD and HFD mice supplemented with or without McPc. (E) Analyses of blood LDL, HDL and total cholesterol. (F) Analyses of blood AST and ALT. The values represent the mean ± SD (n=7); *P<0.05, **P<0.01 and ***P<0.001. ns=not significant. One-way ANOVA Tukey’s post hoc test was performed for statistical analysis.
