# Supplementary material for: Effects of Fermented Polygonum cuspidatum on the Skeletal Muscle Functions
Source: Nutrients. 2024 Jan 19;16(2):305. doi: 10.3390/nu16020305 (PMC10818974; doi:10.3390/nu16020305)
Supplement: Supplementary file 1 [file nutrients-16-00305-s001.zip › Supplementary Table (Table S1, Table S3)_2785447-R1.docx]

**Table S1. Amplicon size and Gene accession numbers of the qRT-PCR primer**

| Gene | Amplicon size(bp) | Accession number |
| --- | --- | --- |
| LPL | 109 | NM_008509 |
| GPX3 | 122 | NM_001329860 |
| NQO1 | 122 | NM_008706 |
| ERRγ | 108 | NM_001243792 |
| ADH1 | 99 | NM_007409 |
| NMRK2 | 108 | NM_027120 |
| ACOT4 | 101 | NM_134247 |
| GSTA2 | 129 | NM_008182 |
| PPARγ | 102 | NM_001127330 |
| PGC1α | 219 | NM_001402987 |
| ERRα | 236 | NM_001413228 |
| TFAM | 117 | NM_009360 |
| HK1 | 140 | NM_001146100 |
| ALDOA | 180 | NM_001177307 |
| ENO1 | 118 | NM_001379127 |
| PDK1 | 122 | NM_001360002 |
| ACSL1 | 106 | NM_001302163 |
| CPT1A | 93 | NM_013495 |
| CPT2 | 203 | NM_009949 |
| NDUFA9 | 128 | NM_025358 |
| SDHB | 104 | NM_001355515 |
| UQCRC2 | 121 | NM_025899 |
| COX5A | 104 | NM_007747 |
| ATP5A1 | 101 | NM_007505 |
| Mstn | 96 | NM_010834 |
| Fbxo32 | 214 | NM_026346 |
| Trim63 | 107 | NM_001039048 |
| β-actin | 112 | NM_007393 |

**Table S3. Information of Antibodies**

| Name of antibody | Manufacturer | Catalogue number |
| --- | --- | --- |
| LPL | Santa Cruz | sc-373759 |
| Total OXPHOS antibody cocktail | Abcam | ab110413 |
| PPARγ | Santa Cruz | sc-7196 |
| p-AMPK | Cell signaling | 2535S |
| p-ACC | Cell signaling | 11818S |
| p-mTOR | Cell signaling | 5536S |
| MyoD | Santa Cruz | sc-377460 |
| Myogenin | Santa Cruz | sc-12732 |
| MyHC | Santa Cruz | sc-376157 |
| α-Tubulin | Santa Cruz | sc-5286 |
